# Supramolecular nanofibers co-loaded with dabrafenib and doxorubicin for targeted and synergistic therapy of differentiated thyroid carcinoma

**DOI:** 10.7150/thno.82140

**Published:** 2023-04-01

**Authors:** Peng Chen, Xiaoyao Cai, Ganen Mu, Yuansheng Duan, Chao Jing, Zhimou Yang, Cuihong Yang, Xudong Wang

**Affiliations:** 1 Department of Maxillofacial and Otorhinolaryngological Oncology, Tianjin Medical University Cancer Institute and Hospital, National Clinical Research Center for Cancer, Key Laboratory of Cancer Prevention and Therapy, Tianjin's Clinical Research Center for Cancer, Tianjin 300060, China.; 2 Key Laboratory of Radiopharmacokinetics for Innovative Drugs, Chinese Academy of Medical Sciences, Tianjin Key Laboratory of Radiation Medicine and Molecular Nuclear Medicine, Institute of Radiation Medicine, Chinese Academy of Medical Sciences & Peking Union Medical College, Tianjin 300192, China.; 3 State Key Laboratory of Medicinal Chemical Biology, College of Life Sciences, Key Laboratory of Bioactive Materials, Ministry of Education, Collaborative Innovation Center of Chemical Science and Engineering, and National Institute of Functional Materials, Nankai University, Tianjin 300071, China.

**Keywords:** supramolecular self-assembly, differentiated thyroid carcinoma, doxorubicin, dabrafenib, BRAF V600E

## Abstract

**Rationale:** Although surgery, radioiodine therapy, and thyroid hormone therapy are the primary clinical treatments for differentiated thyroid carcinoma (DTC), effective therapy for locally advanced or progressive DTC remains challenging. BRAF V600E, the most common BRAF mutation subtype, is highly related to DTC. Previous studies prove that combination of kinase inhibitors and chemotherapeutic drugs may be a potential approach for DTC treatment. In this study, a supramolecular peptide nanofiber (SPNs) co-loaded with dabrafenib (Da) and doxorubicin (Dox) was constructed for targeted and synergistic therapy with BRAF V600E^+^ DTC.

**Methods:** A self-assembling peptide nanofiber (Biotin-G^D^F^D^F^D^YGRGD, termed SPNs) bearing biotin at the N-terminus and a cancer-targeting ligand RGD at the C-terminus was used as a carrier for co-loading Da and Dox. D-phenylalanine and D-tyrosine (^D^F^D^F^D^Y) are used to improve the stability of peptides *in vivo*. Under multiple non-covalent interactions, SPNs/Da/Dox assembled into longer and denser nanofibers. RGD ligand endows self-assembled nanofibers with targeting cancer cells and co-delivery, thereby improving cellular uptake of payloads.

**Results:** Both Da and Dox indicated decreased IC50 values upon encapsulation in SPNs. Co-delivery of Da and Dox by SPNs exhibited the strongest therapeutic effect *in vitro* and* in vivo* by inhibiting ERK phosphorylation in BRAF V600E mutant thyroid cancer cells. Moreover, SPNs enable efficient drug delivery and lower Dox dosage, thereby significantly reducing its side effects.

**Conclusion:** This study proposes a promising paradigm for the synergistic treatment of DTC with Da and Dox using supramolecular self-assembled peptides as carriers.

## Introduction

Although the overall prognosis of thyroid cancer is adequate, approximately 30% to 80% of patients with papillary thyroid cancer (PTC) develop cervical lymph node metastasis, and approximately 10% develop distant metastasis 1, 2. Patients with strong invasiveness invariably have poor prognosis. Studies have indicated that 5-year survival rate of patients with IV-stage thyroid cancer is only 50%, and the recurrence rate is as high as 20% to 40% 3, 4. Currently, differentiated thyroid carcinoma (DTC) is treated effectively with surgery, thyroid stimulating hormone (TSH) suppressive therapy and radioiodine therapy (RAI) 5, 6. However, effective treatment of patients with local recurrence and/or distant metastasis remains challenging due to the inadequate effects of conventional treatment 7-9. V-Raf murine sarcoma viral oncogene homolog B (BRAF) is an important human proto-oncogene that encodes the RAF family serine/threonine protein kinases. BRAF mutations are related to approximately 8% of malignant tumors. BRAF V600E is the most common BRAF mutation subtype among various tumors 10. The positive rate of BRAF V600E in DTC is 30% to 70%, which is highly related to lymph node metastasis and poor prognosis, including tumor recurrence, absence of ^131^I avidity, and treatment failure 11. Previous studies have demonstrated that a combination of kinase inhibitors and chemotherapeutic drugs can achieve synergistic antitumor effects 12, 13. For example, recent clinical trials have demonstrated that the co-administration of sorafenib and doxorubicin significantly improves the overall survival rate 14. Thus, based on this encouraging evidence, synergistic therapy with kinase inhibitors and chemotherapeutic drugs may be a potential approach for DTC treatment.

Currently, several BRAF V600E inhibitors, such as vemurafenib and dabrafenib, have been used to treat melanoma, thyroid cancer, and colorectal cancer, and have attained a certain therapeutic effect. Doxorubicin (Dox) remains the most effective cytotoxic chemotherapy regimen for patients with high-risk metastatic DTC who are in progress after the standard treatment 15. Accordingly, in view of the limited treatment options for radioiodine-refractory DTC patients and its close correlation with BRAF V600E high expression, the feasibility of combination treatment of BRAF V600E inhibitors with Dox for BRAF V600E overexpressed DTC is worthy of further study. However, these clinical drugs for refractory DTC therapy usually fail to exert the desired effects owing to their poor water solubility, rapid elimination, inadequate bio-stability, and undesired toxicity 16. Some kinase inhibitors induce resistance through complex mechanisms 17, 18. Additionally, the efficacy of combination treatments is hampered by their distinct physicochemical profiles. Therefore, the design of targeted and effective carrier systems is highly significant for the safe and efficient combination of Dox and BRAF V600E inhibitors for DTC therapy.

Nanomaterials have been extensively developed and employed as promising vehicles for efficient drug delivery, which can significantly enhance the therapeutic efficacy of drugs and reduce undesired side effects by precisely delivering and releasing drugs to the target tissues 19, 20. In this regard, self-assembled peptides show significant potential for targeted drug delivery and disease diagnosis and treatment because of their remarkable advantages, such as flexible structure design, readily targeted modification, high biosafety, and ability to assemble with a variety of drugs to form nanostructures 21. Nano-drugs have gradually been used in the diagnosis and treatment of TC, exhibiting good efficacy 22, 23. Although self-assembled peptides have rarely been adopted as a delivery platform in thyroid cancer therapy, various convincing evidences demonstrate that they may be promising for the treatment of inoperable or metastatic thyroid cancer 24-26.

**Figure 1:**
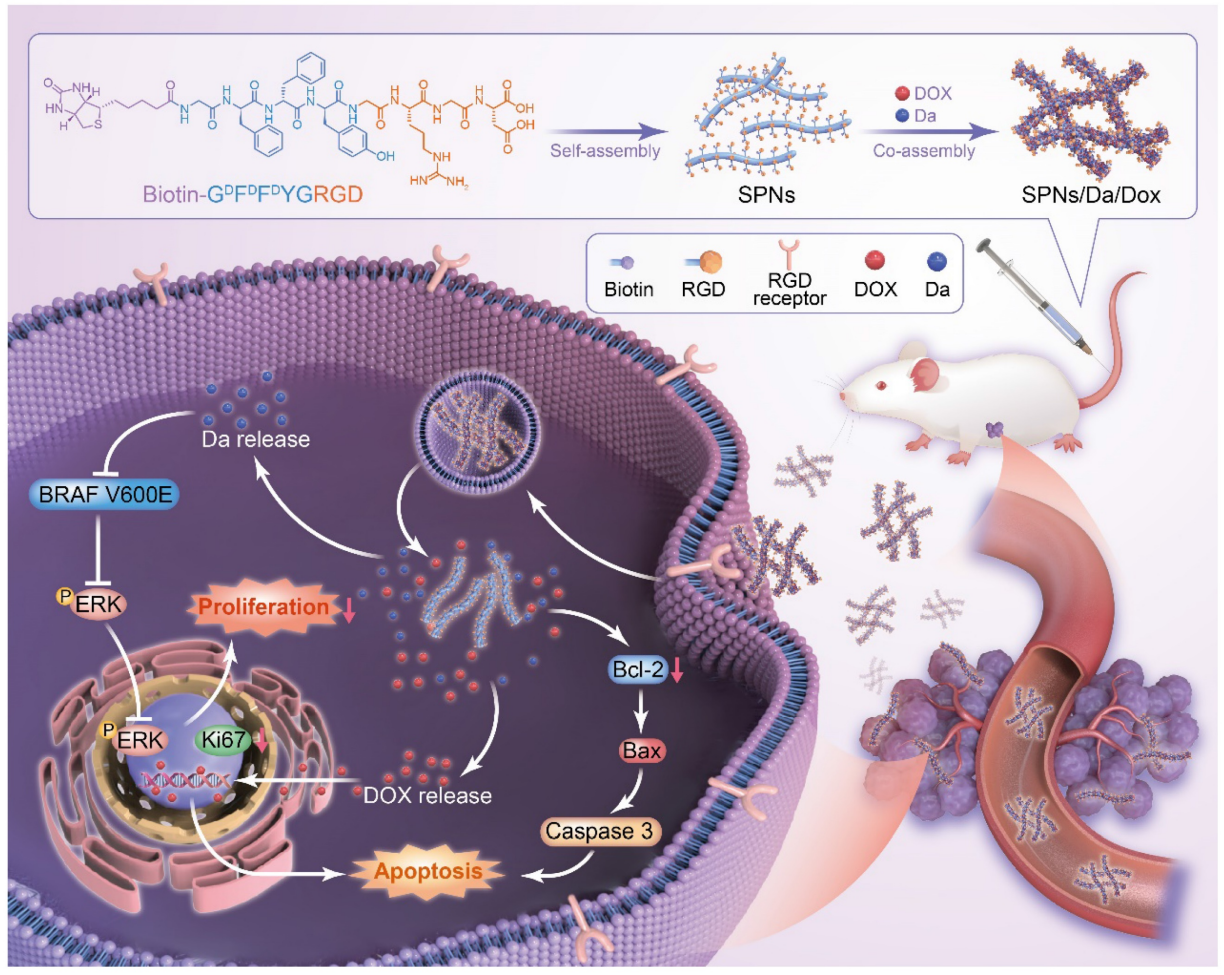
Schematic of co-delivery of dabrafenib (Da) and doxorubicin (Dox) by self-assembly peptide nanofibers for treatment of BRAF V600E mutant differentiated thyroid carcinoma. By means of non-covalent interactions, Da and Dox are co-assembled with the supramolecular nanofibers. Nanofibers can actively target cancer cells through RGD ligand and be internalized, after which Da and Dox are sustainedly released from the nanofibers to exert synergistic antitumor effects by inhibiting cancer cell proliferation and promoting cell apoptosis.

Herein, a supramolecular peptide nanofiber (SPNs) co-loaded with dabrafenib (Da) and Dox (named SPNs/Da/Dox) was constructed for targeting and synergistic therapy of thyroid carcinoma. As shown in Figure 1, a self-assembling peptide (Biotin-G^D^F^D^F^D^YGRGD, termed SPNs) bearing biotin at the N-terminus and a cancer-targeting ligand RGD at the C-terminus was designed. Because biotin ligands are highly expressed in various cancer cells, the introduction of biotin can promote the binding of assembled nanofibers to cancer cells as well as provide non-covalent interactions for assembly. To improve the stability of peptides *in vivo*, D-phenylalanine and D-tyrosine (^D^F^D^F^D^Y) are employed. In the presence of RGD ligands, self-assembled nanofibers can target cancer cells, co-deliver, and improve the specific cellular uptake of payloads. Owing to the existence of multiple benzene rings and non-polar properties, Da and Dox are involved in the process of molecular self-assembly, resulting in the formation of nanofibers co-loaded with the two drugs (SPNs/Da/Dox). Da released from SPNs/Da/Dox can specifically inhibit BRAF V600E mutant cancer cells because it inhibits the MAPK signaling pathway by reducing the cytoplasmic level of ERK phosphorylation (p-ERK). Dox-targeted delivery to cancer cells can induce apoptosis more efficiently than free drug. Therefore, relying on the supramolecular nanoplatform, Da and Dox are targeted and co-delivered to thyroid cancer, exerting excellent synergistic antitumor effects by cooperatively inhibiting cancer cell proliferation and promoting cell apoptosis.

## Materials and Methods

### Materials

2-chlorotrityl chloride resin, Fmoc-Asp (OtBu)-OH, Fmoc-Dopa-(acetonide)-OH, Fmoc-Arg(pbf)-OH, Fmoc-Gly-OH, Fmoc-Tyr-OH (D configuration), and Fmoc-Phe-OH (D configuration) were purchased from Shanghai GL Biochem (Shanghai, China). Biotin and Dox were provided by Energy Chemical Co. Ltd. (Shanghai, China). Dabrafenib was purchased from Medmol (Italy). Other general chemical reagents, solvents, and common materials were purchased from commercial sources without further purification unless otherwise noted. Dulbecco's modified Eagle medium (DMEM) was obtained from Gibco (USA). Fetal Bovine Serum (FBS) and Phosphate buffer saline (PBS) was obtained from BI (Israel). Penicillin-sodium and streptomycin were purchased from HyClone (USA). 3-(4, 5-Dimethylthiazol-2-yl)-2, 5-diphenyltetrazolium bromide (MTT) was provided by Heowns Co. Ltd. (Tianjin, China). The apoptosis assay kits were purchased from Keygen Biotech Co. Ltd. (Nanjing, China). Hoechst 33342 was obtained from Beyotime Co. Ltd. (Shanghai, China). LysoTracker Red DND-99 was purchased from Invitrogen (UK). BCPAP, 8505C, CAL-62, TPC-1, and L02 cells were maintained in our laboratory and cultured in DMEM with 10% FBS and 1% penicillin/streptomycin. BRAF V600E and Tubulin antibodies were purchased from Abcam (Cambridge, UK). Ki67 and Bcl-2 antibodies were purchased from Zhongshan Goldbridge Biotechnology (Beijing, China). Antibodies against Bax, t-ERK, and p-ERK were purchased from Proteintech Group, Inc. (USA). The Cleaved-Caspase 3 antibody was purchased from Affinity Biosciences (USA). Na^125^I was purchased from PerkinElmer (Canada). ^1^H NMR spectra were recorded on a Bruker 400M spectrometer using Methanol-d_4_ as the solvent (Germany). High-resolution mass spectrometry (HR-MS) was conducted on a 6520 Q-TOF LC/MS using an ESI-L low-concentration tuning mix (USA). Transmission electron microscopy (TEM) images were recorded on an HT7700 Exalens instrument (Japan). Rheological tests were performed on an Anton Paar MCR302 (Austria). Dynamic light scattering (DLS) was performed using a BI-200SM and BI- 9000AT (USA). Ultraviolet-visible spectroscopy was performed using a UV-2450 UV-vis spectrophotometer (Japan). Cell viability and absorption intensity were recorded using a Synergy 4 (USA). The circular dichroism (CD) spectra were recorded using a BioLogic MOS-450 (France). Western blotting was performed using a Bio-Rad (USA). Flow cytometry was performed using BD LSR Fortessa (USA). Cell uptake and subcellular localization images were acquired using confocal laser scanning microscopy (Leica, Germany). Radioactive isotope imaging was carried out using Gamma camera (KODAK IS *in vivo* FX, USA).

### Peptide synthesis

Biotin-G^D^F^D^F^D^YGRGD was synthesized from the corresponding amino acids using the standard Fmoc solid-phase peptide synthesis (SPPS) technique with the application of 2-chlorotrityl chloride resin and the corresponding N-Fmoc-protected amino acids with side chains. First, 0.5 g of 2-chlorotrityl chloride resin was swelled in dry dichloromethane (DCM) with nitrogen (N_2_) bubbling for 30 min and then washed five times with dry dimethylformamide (DMF). Further, the first amino acid was solubilized in DMF with *N,N*-diisopropylethylamine (DIEA) and conjugated to the resin at the C-terminus of the amino acid. After reacting for 1.5 h, the resin was rinsed with dry DMF five times and quenched by a blocking solution (17:2:1 of DCM/methanol/DIEA) for 10 min twice. Then, the N-Fmoc protected group in the resin was removed by treatment with 20% piperidine in DMF for 30 min and washed thoroughly with DMF five times. O-(Benzotriazol-1-yl)-*N,N,N',N*'-tetramethyluronium hexafluorophosphate (HBTU) was used as a coupling reagent to couple the next amino acid to the free amino group. Subsequently, the peptide chain was extended according to the standard SPPS protocol. Afterwards, the synthesized peptide was cleaved from the resin using 95% trifluoroacetic acid (TFA) containing 2.5% H_2_O and 2.5% TIS, and the mixture was refined by rotary evaporation. The crude products were isolated by precipitation in cold diethyl ether, and subsequently dried using a vacuum pump. Finally, the crude peptides were purified by HPLC using MeOH and H_2_O containing 0.05% of TFA as eluents and lyophilization to gain the pure peptides.

### Preparation of SPNs and different drug formulations

SPNs (0.1 wt%) were prepared by dissolving 1.0 mg of Biotin-G^D^F^D^F^D^YGRGD and 2 equiv of Na_2_CO_3_ (to adjust the pH to 7.4) in 1.0 mL of PBS (pH = 7.4) via a heating-cooling process. The preparation of SPNs/Da, SPNs/Dox, and SPNs/Da/Dox was performed using the following procedure. Briefly, both Da and Dox were dissolved in DMSO at the indicated concentrations. Subsequently, Da, Dox, and Da/Dox were added to the prepared compound solution at a mass ratio of 5% and a volume ratio of 2.5% (free agents: peptide = 1:20) before the heating-cooling process, and followed by ultra-sonification for 30 min, respectively. Finally, SPNs/Da, SPNs/Dox, and SPNs/Da/Dox were collected after sitting overnight at room temperature via a heating-cooling process. And nanofiber solutions were prepared by diluting hydrogel with PBS to corresponding concentration for *in vitro* and *in vivo* experiments.

### Rheological Test

The rheology tests were carried out using the Anton Paar MCR302 system at the temperature of 37 °C. Parallel plates (25 mm) were used during the experiment with a gap of 500 μm. The elastic modulus (G') and viscosity modulus (G”) of samples were then conducted in the frequency region of 0.1-100 rad/s at a strain of 1%.

### Determination of critical assembly concentration (CAC)

To study the assembling ability of the peptides, the critical aggregation concentration (CAC) was determined by dynamic light scattering (DLS) using a laser light scattering spectrometer (BI-200SM) equipped with a digital correlator (BI- 9000AT) at 532 nm at room temperature (22‒25 °C). All containers were washed with acetone and dried under vacuum to eliminate dust, and the PBS solvent was filtered through a 0.22 μM sterile membrane prior to the measurement. Briefly, 100 μL of samples were placed in a specific dish, and the light scattering intensity of different concentrations of samples was optimized and recorded by 90 PLUS. The CAC values were analyzed and fitted using Origin 9.6. Lower CAC values represent a better assembly capacity of the sample.

### Transmission Electron Microscope (TEM) characterization

A negative staining technique was used to observe the TEM images of the microstructures of the samples. 10 μL of samples were placed on a carbon-coated copper grid, and phosphotungstic acid was then used for negative staining. After air-drying overnight in a desiccator, the nanostructures were observed under a transmission electron microscope (Hitachi HT7700) operating at 200 kV.

### Circular dichroism (CD) spectrum

The CD spectrum was obtained using a BioLogic (MOS-450) system. After subtracting the solvent background, all samples were loaded onto 0.1 cm quartz slides at 25 °C. The resultant CD spectrum was acquired by setting the conditions as follows: the wavelength ranged from 180 to 260 nm, the acquisition period was 0.5 s, and the step was 0.5 nm.

### *In vitro* Dox solubility

The solubility of the samples was determined by UV-vis spectroscopy. Briefly, the standard curve represents the relationship between the concentrations of Dox in the solutions, and the corresponding absorbance was pre-plotted. The solubility of Dox (0.5 mM) co-assembled with SPNs at different concentrations was determined using a standard curve. Different concentrations of Dox co-assembled with SPNs (0.1% wt) were subsequently determined by measuring the absorbance at 480 nm via UV-vis spectroscopy. The experiment was performed in triplicate, and the data are presented as mean ± SD.

### *In vitro* cellular uptake

Cellular uptake was estimated using flow cytometry. BCPAP cells were seeded in 6 well-plates at a density of 3×10^5^ cells per well and cultured for 24 h in an atmosphere of 5% CO_2_ at 37 °C. Then, the treatments described above were incubated with cells in 1 mL fresh serum-free DMEM medium at a Dox concentration of 0.5 µM for 4 and 12 h. After incubation, the cells were gently trypsinized, collected, and thoroughly washed three times with cold PBS. Finally, the resuspended cells were incubated in 500 μL of PBS and counted using flow cytometry.

### *In vitro* cytotoxicity assay

The cytotoxicity of peptides, free agents, and the co-assemblies towards BCPAP and L02 cells was evaluated using the standard MTT assay. Briefly, the cells were seeded into 96-well plates at a density of 8×10^3^ cells per well in 100μL of DMEM medium containing 10% FBS and 1% penicillin-streptomycin. After overnight pre-incubation, the cells were treated with the compound, free agents, and co-assemblies at different concentrations for 48 h. Untreated tumor cells were used as controls. Subsequently, the medium was replaced with an MTT solution (100 μL, 0.5 mg/mL) and further cultured for 4 h. Finally, the supernatant was removed and 100 μL of DMSO was added to each well to dissolve the purple crystals. Cell viability was measured using a microplate reader by recording the absorbance of the formazan product at 570 nm.

### Endocytosis studies

BCPAP cells in DMEM were seeded in a confocal glass-bottom cell culture dish at a density of 1.0×10^5^ cells per dish. After incubation for 24 h, the DMEM solution containing the treatments at a Dox concentration of 2.5 µM was added to the cells for 1, 2, 4, and 8 h. The treatment solution was removed, and the cells were washed three times with PBS. Cells were first stained with 50 nM of LysoTracker Red DND-99 for 15 min at 37 °C in the dark. The cells were then rinsed thrice with PBS. Cells were then stained with 1.0 μg/mL of Hochest 33342 for 10 min at 37 °C in dark. Finally, we recorded the images using laser scanning confocal microscopy (E_x_ = 561 nm for the red channel; E_x_ = 488 nm for the yellow channel; E_x_ = 405 nm for the blue channel). All images were obtained using a laser scanning confocal microscope (Leica TSC SP8) at the same voltage.

### Cell apoptosis analysis

Apoptosis in BCPAP cells was measured using an Annexin V-APC/7-AAD Apoptosis Detection Kit. Briefly, BCPAP cells in DMEM were seeded into 6-well plates at a density of 3×10^5^ cells per well and incubated overnight. After treatment with PBS and Dox concentration of 0.5 µM for 36 h, the cells were trypsinized, collected, washed twice with cold PBS, and centrifuged at 1500 rpm for 5 min. The precipitated cells were resuspended in 500 μL Annexin V-APC binding buffer and incubated with 5 μL Annexin V-APC and 5 μL 7-AAD at room temperature for 15 min in the dark. The samples were then analyzed using a flow cytometer.

### Western blot analysis

The indicated cells (3×10^5^/well) were seeded in 6-well plates and cultured in DMEM overnight. Cells were then treated with Da and SPNs/Da at the Da concentration of 0.5 µM for 0, 6, 12 h. Additionally, cells were treated with 1) PBS, 2) Da, 3) Dox, 4) Da/Dox, 5) SPNs/Da, 6) SPNs/Dox and 7) SPNs/Da/Dox at the Da concentration of 0.5 µM for 6 h. Subsequently, the cells were lysed in prechilled cell lysis buffer containing protease and phosphatase inhibitor cocktail. The concentration of protein lysates was determined by the BCA protein assay and subjected to western blot analysis. Equal amounts of protein lysates were separated by SDS sulfate-polyacrylamide PAGE and transferred onto PVDF membranes. The membranes were then incubated overnight at 4 °C with individual primary antibodies, followed by incubation with species-specific horseradish peroxidase (HRP)-conjugated secondary antibodies. Finally, the signals were visualized using ECL luminescence detection reagent and western blotting detection system.

### *In vitro* biostability

For *in vitro* biostability study, plasma was prepared by collecting whole blood of healthy mice into heparinized tube and centrifugation at 3500 rpm for 10 min. SPNs were incubated in plasma at concentration of 0.5 mg/mL at 37 °C for 6 h, 12 and 24 h, respectively. For sample preparation, 4-fold volume of acetonitrile was added to the incubated plasma sample, vortexed, and centrifugated at 12000 rpm at 4 °C for 10 min to sediment the protein impurities. To quantitatively study the degradation rate of the SPNs in the blood environment, the samples were also analyzed by LC-MS.

### *In vivo* radioactive imaging of ^125^I-labelled SPNs

Na^125^I (0.5 mCi) in 10 mM PBS was added to SPNs solution in PBS (1 mg/mL). The reaction was performed at 20 °C for 5 min. Then, 100 μL of sodium peroxodisulfate was added to terminate the reaction. The unreacted ^125^I and other chemicals were removed by radioactive HPLC (Dionex UltiMate3000, America). The mobile phase was 10% deionized water and 90% ethanol. The radiochemical purity of the peptides was detected by radioactive thin-layer chromatography (TLC) scanner (BioScan, America). Then, BCPAP cells (5×10^6^ cells) were resuspended in 100 μL PBS and injected subcutaneously into the left flank of the NSG mice. In order to verify the blocking effect of NaI on the sodium/iodide symporter, NaI and Na^125^I were sequentially intravenously injected when the tumor volume reached 150-200 mm^3^. Afterwards, for biodistribution assay, NaI and ^125^I-labeled SPNs (1 mg/mL) were sequentially intravenously injected when the tumor volume reached 150-200 mm^3^ (n = 4). The mice were anesthetized and imaged by planar Gamma camera at 0.5, 2, 8, 24 h post injection.

### *In vivo* antitumor activity

All animal experiments were performed according to the protocol approved by the Animal Care and Use Committee of the Tianjin Medical University Cancer Institute and Hospital. NSG mice (5 weeks, female) were purchased from Gempharmatech Co., Ltd. (Jiangsu, China). BCPAP cells (5×10^6^ cells) were resuspended in 100 μL PBS and injected subcutaneously into the left flank of the mice. Once the tumor volume reached 50-75 mm^3^, the tumor-bearing mice were randomly divided into 7 groups (n = 4) and intravenously injected with 200 μL of different formulations every four days for five times as follows:1) PBS (Control), 2) Da, 3) Dox, 4) Da/Dox, 5) SPNs/Da, 6) SPNs/Dox and 7) SPNs/Da/Dox, with a dose of 0.5 mg/kg for Dox or with a dose of 0.5 mg/kg for Da. The body weight and tumor size of each mouse were monitored and recorded every two days during the treatment process of 20 days. The tumor volume (V) was measured with a digital caliper and calculated using the equation: V= (L×W^2^)/2, where W and L refer to the longer and shorter diameters of the tumors, respectively. At the end of the experiment, the mice were euthanized, and the tumor tissues and main organs (heart, liver, spleen, lung, and kidney) were photographed, weighed, and collected. Furthermore, the collected *ex vivo* tumors and main organs were fixed with 4% paraformaldehyde for the subsequent hematoxylin and eosin (H&E), terminal deoxynucleotidyl transferase-mediated deoxyuridine triphosphate nick end labeling (TUNEL), and immunohistochemical staining, including Ki67, BRAF V600E, p-ERK, Bcl-2, Bax and Cleaved-Caspase 3. Blood was obtained from the mouse eye socket for blood biochemistry and routine blood analysis, which was compared with the blank group without tumor inoculation.

### Statistical analyses

The data are expressed as mean ± standard deviation. Statistical analyses were performed using analysis of variance for multiple comparisons and Students' t-test for two-group comparisons. All experiments were performed in triplicates. Statistical significance was set at * *P* < 0.05, ** P < 0.01, *** P < 0.001 and **** *P* < 0.0001.

## Results and Discussion

### Preparation and characterization of nanoformulations

**Figure 2:**
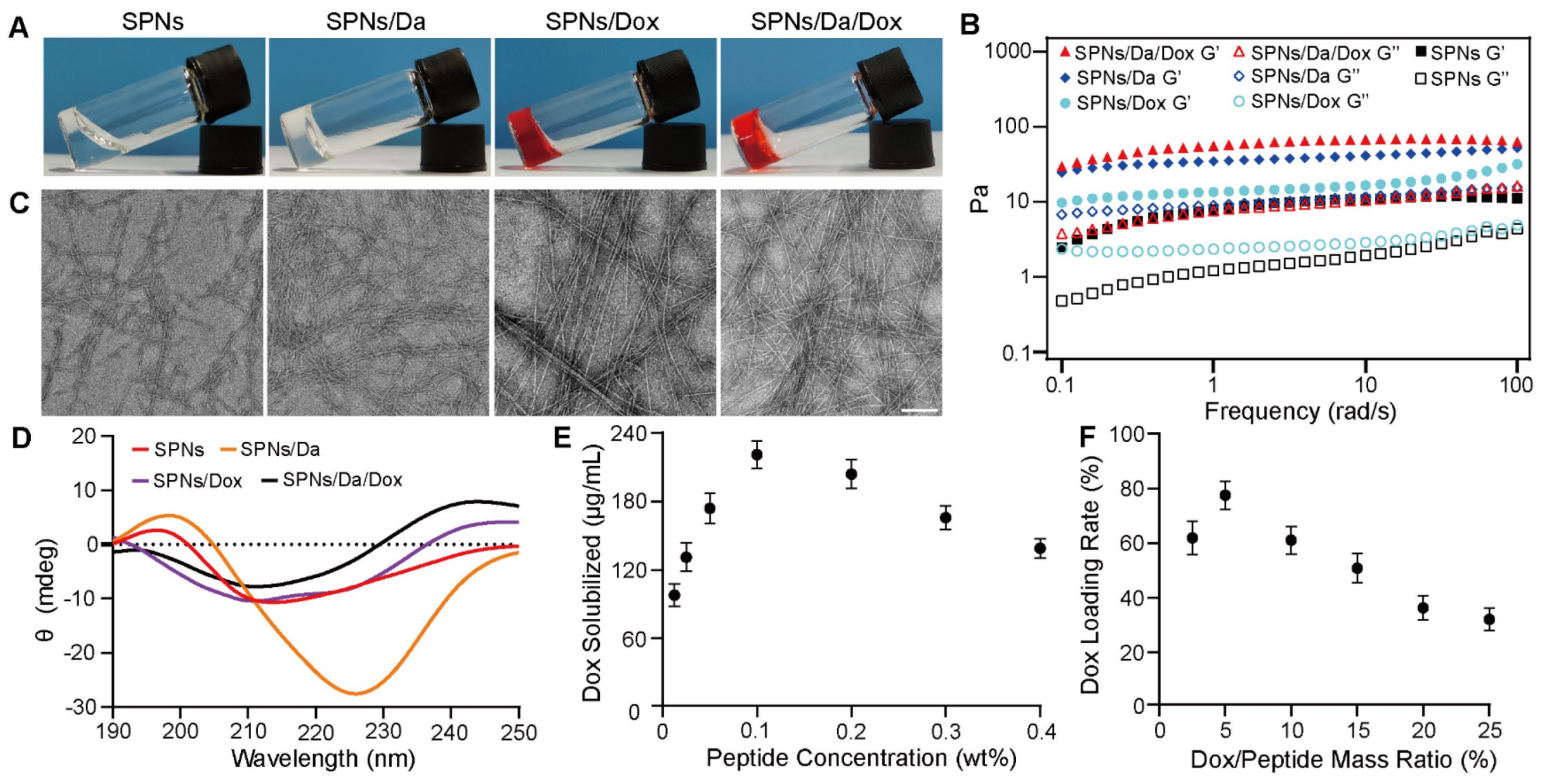
Characterization and drug loading behaviors of nanoformulations. (A) Optical images of SPNs, SPNs/Da, SPNs/Dox and SPNs/Da/Dox with the peptide concentration of 0.1 wt% after heating-cooling process. (B) Rheological properties of different hydrogels in the frequency region of 0.1-100 rad/s at a strain of 1%. Solid and hollow symbols represent storage moduli (G') and loss moduli (G”) respectively. TEM images (C) and circular dichroism spectra (D) of assemblies. The scale bar was 100 nm. (E) The loading concentration of Dox under different peptide concentrations with fixed Dox concentration of 0.5 mM. (F) Loading efficiency of SPNs (0.1 wt%) with different Dox/SPNs mass ratios. The data in (E) and (F) represent the means ± SD (n = 3).

To prepare a self-assembling peptide system for the co-delivery of Da and Dox, Biotin-G^D^F^D^F^D^YGRGD (SPNs) was synthesized by standard solid-phase peptide synthesis (Figures S1 and S2). ^1^H NMR and mass spectrometry analyses confirmed the successful synthesis (Figures S3 and S4). After the successful synthesis of the SPNs, their assembling ability was tested. The SPNs exhibited good solubility in PBS (pH = 7.4), forming a clear solution at a concentration of 0.1 wt%. A weak gel was formed after the heating-cooling process (Figure 2A). The Dyndall effect was observed after the heating and cooling processes, which also indicates the self-assembly ability of the prepared peptide (Figure S5).

The co-assembly ability of SPNs with Dox and Da was tested. Figure 2A shows that compared with SPNs, co-assembly with either 5% w/w Da, Dox, or mixture of Da and Dox (molar ratio = 1:1) could produce hydrogels with higher mechanical strength. Rheological data confirmed these results (Figure 2B). For all groups, the elastic modulus (G') values were larger than the corresponding viscosity modulus (G”) values, indicating hydrogel formation. In addition, both Da and Dox improved the mechanical properties of the resulting hydrogels. Meanwhile, the G' of the stable hydrogel formed by SPNs in the presence of Da and Dox was approximately 10-fold higher than that of the hydrogel formed by SPNs alone. The results of critical assembly concentration (CAC) analysis (Figure S6) shows that the CAC value of the SPNs was 140.5 μM. When co-assembled with 5% w/w Da or Dox, the CAC value decreased to 68.4 μM and 110 μM, respectively. Remarkably, SPNs/Da/Dox had the lowest CAC (57.5 μM), indicating that Da and Dox could enhance the assembly of SPNs. This may be due to the involvement of multiple benzene rings of Dox and Da in π-π interactions in the process of SPNs assembly.

To further analyze the micromorphology of the different assemblies, transmission electron microscopy (TEM) analysis (Figure 2C) was performed. After the heating-cooling process, the SPNs were characterized as short and sparse fibers, which may be the reason for the formation of a relatively weak hydrogel by the SPNs. After co-assembly with Da, the fiber length was prolonged, and the fiber network was denser. In contrast, longer and straighter fiber bundles were formed after co-assembly with Dox. The fibers of the assemblies formed by the co-assembly of SPNs with both Da and Dox were similar to those of the co-assemblies of SPNs with Dox. The CD spectra was shown in Figure 2D. Nanofibers of SPNs had positive and negative peaks at 196 nm and 212 nm respectively, which was the hallmark of β-sheet. After the addition of Da, the peak red shifted and the peak intensity increased, which may be due to the fact that the π-π stacking between aromatic-rich Da and peptide led to a more extensive conjugated system. Conversely, after the addition of Dox, the secondary structure was similar to α-Helix, positive peak at 196 nm and negative peak at 212 nm and 225 nm, possibly because the Dox containing oxygen and nitrogen atoms interacted more with peptide main chain through hydrogen bond, which changed molecular alignment 27. Furthermore, nanofibers co-loaded with Da and Dox exhibited a non-typical and mixed molecular conformation, probably resulting from the complex synergistic effects of π-π stacking and hydrogen bond between drug and peptide. The UV-vis absorption spectrum was used to determine the encapsulation ability of Dox with different concentrations of SPNs and the loading efficiency of Dox under different Dox and SPNs feed ratios (Figures S7, S8 and S9). Results in Figure 2E shows that, when the concentration of SPNs was 0.1 wt%, the loading capacity of Dox was the strongest, which was 221.9 μg/mL. When the mass ratio of Dox/SPNs was 5%, the loading rate of Dox was up to 77.8% (Figure 2F). Accordingly, the 0.1 wt% of SPNs and 5% mass ratio of Dox/SPNs were chosen for the following assays. These results indicate that Da and Dox can co-assemble with SPNs to form nanofibers that can net a large number of water molecules and eventually form hydrogels with better mechanical properties.

### Cellular uptake and specificity of nanoformulations

**Figure 3:**
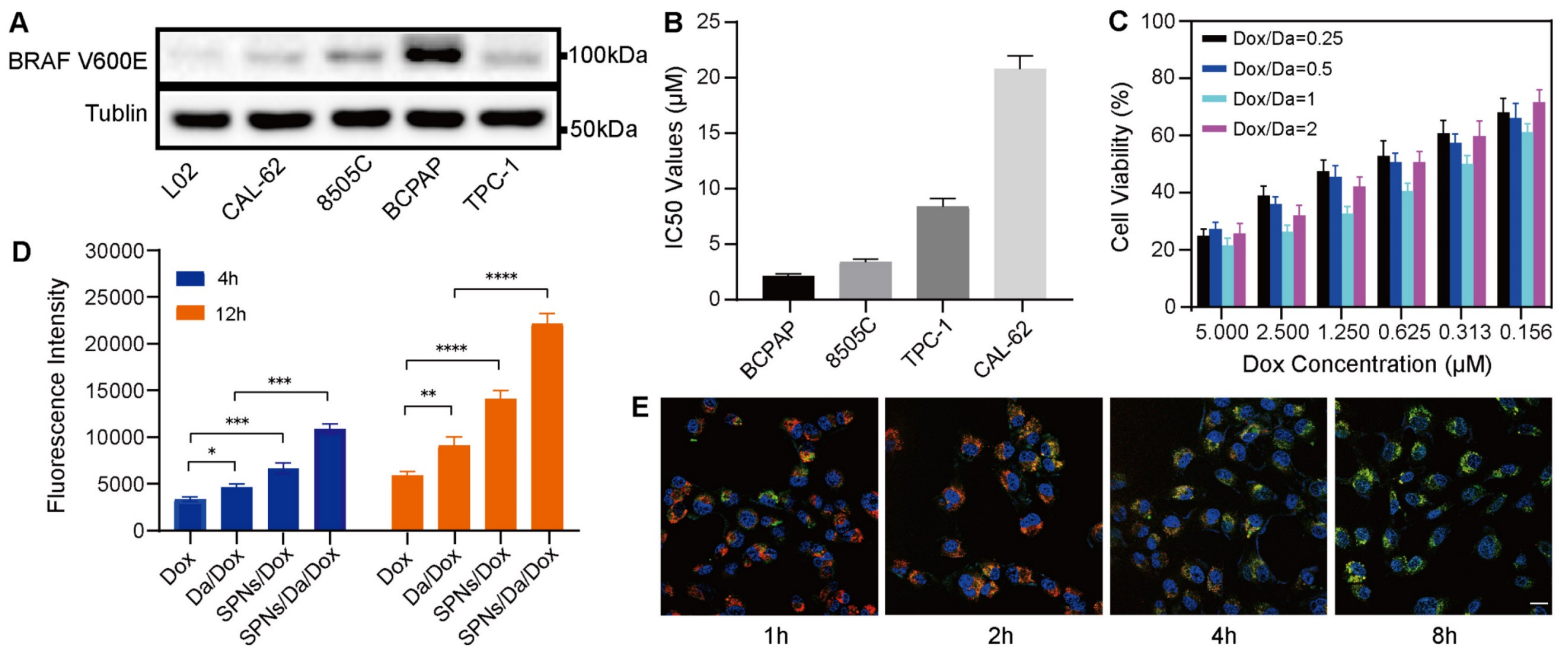
BRAF V600E specific killing and cellular uptake of nanoformulations. (A) Western blot analysis of BRAF V600E expression in different cell lines. (B) IC50 values of Da towards different cell lines (n = 3). (C) Cytotoxicity of SPNs/Da/Dox with SPNs concentration of 0.1 wt% and different mass ratios of Dox and Da (1:4, 1:2, 1:1, 2:1) in BCPAP cells (n = 3). (D) Detection of Dox fluorescence in BCPAP cells treated with Dox, Da/Dox, SPNs/Dox and SPNs/Da/Dox for 4 h and 12 h by flow cytometry (*P < 0.05, **P < 0.01, ***P < 0.001, ****P < 0.0001, n = 3). (E) CLSM images of BCPAP cells treated with SPNs/Dox for 1, 2, 4 and 8 h (scale bar = 25 µm).

The efficient cellular uptake of anticancer agents is the premise for achieving anticancer effects. The expression of BRAF V600E in thyroid carcinoma-related cell lines was first screened using western blotting. As shown in Figure 3A, human thyroid cancer papillary (BCPAP) cells significantly overexpressed BRAF V600E compared to other thyroid cancer cells, which is consistent with the results of previous studies 28, 29. The cytotoxicity of Da is mainly attributed to its specificity for the mutant BRAF V600E. Accordingly, a methyl thiazolyl tetrazolium (MTT) assay was used to detect the killing effects of Da on different thyroid cancer cells. The results (Figure 3B) indicated that Da exerted the most significant killing effect on BCPAP cells that had the highest BRAF V600E expression. To further verify the specificity of Da, the cytotoxicity of Da in normal hepatocytes (L02) and BCPAP cells was investigated. The results showed that Da could be more efficiently against BCPAP than L02 (Figure S10).

Subsequently, to study the best co-assembly ratio of Da and Dox in the nanofoumulation, two drugs were loaded into 0.1 wt% SPNs with different mass ratios, and their killing efficacy towards BCPAP cells was studied by MTT assay. The synergistic effect was particularly obvious when the mass ratio of Da to Dox was 1:1, which showed the highest cytotoxicity at all drug concentrations (Figure 3C). Accordingly, a Da/Dox mass ratio of 1:1 was selected for the construction of SPNs/Da/Dox coassemblies in subsequent studies. The cellular uptake behaviors of nanoformulations and free Dox were investigated by incubating BCPAP cells and analyzed by flow cytometry. Quantitative analysis of Dox uptake via flow cytometry showed that SPNs/Dox and SPNs/Da/Dox exhibited more Dox uptake than the free drug group after incubation for 4 and 12 h. Moreover, with the extension of time to 12 h, the cellular uptake of Dox increased in all groups (Figures 3D, S11, and S12). More importantly, BCPAP cells incubated with SPNs/Da/Dox exhibited more than 2.1 times higher fluorescence intensities than the cells treated with free Dox, suggesting a much higher cellular uptake efficiency of targeting nanofibers than free agents. This phenomenon may be due to the presence of multiple integrin-binding peptide ligands (RGD) on the surface of SPNs, which can promote targeting, interaction with cancer cells, and internalization of nanomaterials 30-32. To study the intracellular distribution of SPNs/Dox, Hoechst 33342 (blue) was used to stain the nucleus of cells, Lyso-Tracker Red was used to stain the lysosomes of cells, and green fluorescence indicated the uptake of Dox. Representative CLSM images in Figure 3E show a good overlay between red fluorescence and green fluorescence within 2 h. However, the co-localization of red fluorescence and green fluorescence decreased significantly at 4 h, and even less at 8 h, indicating the lysosomal escape ability of SPNs/Dox 33, 34. No significant lysosomal fluorescence was observed at 8 h. The above results indicate that SPNs have a superior ability for cellular drug delivery and can be a promising carrier system for combined cancer drug delivery.

### *In vitro* synergistic cell inhibition

**Figure 4:**
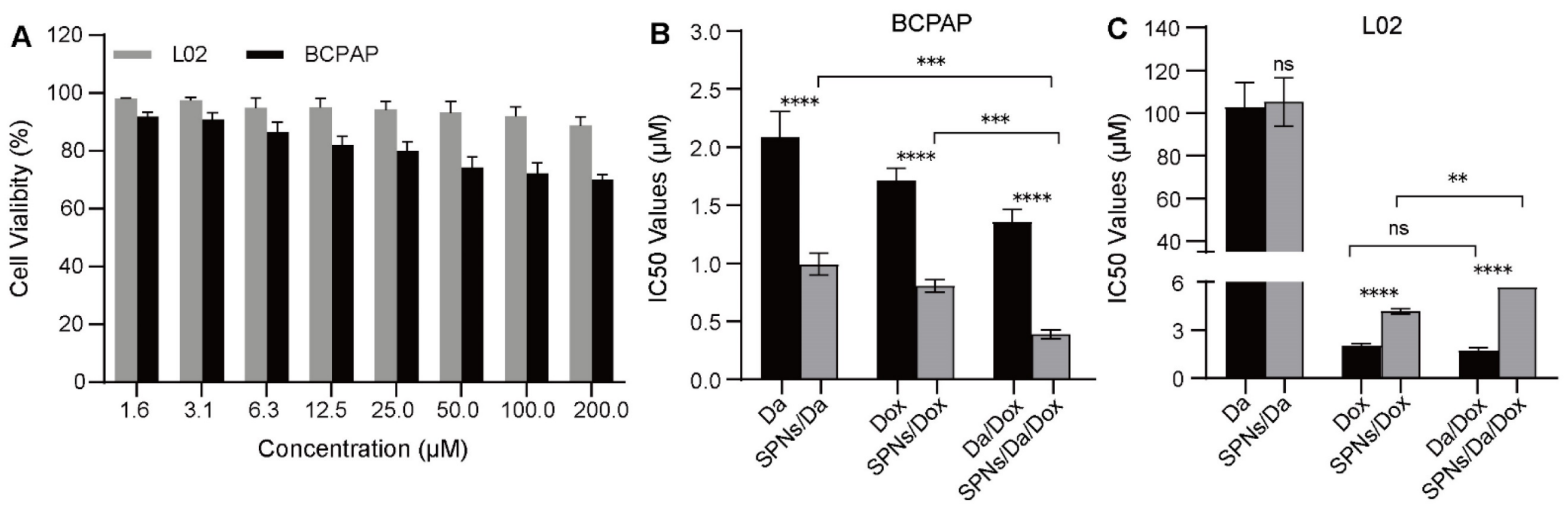
Cellular toxicity of different formulations. (A) Cell viability of BCPAP and L02 cells after incubation with SPNs (1.6-200 μM) for 48 h (n = 3). IC50 values of different formulations on (B) BCPAP cells and (C) L02 cells after incubation for 48 h (**P < 0.01, ***P < 0.001, ****P < 0.0001, n = 3).

The cellular viability of SPNs was examined using an MTT assay. As shown in Figure 4A, after 48 h of incubation, SPNs at low concentrations showed relatively low toxicity, especially to L02 cells. This suggests high biocompatibility and safety of the prepared peptide drug delivery system. The *in vitro* anticancer effects of free Da, Dox, and the corresponding nanoformulations were further evaluated. The mass ratios of the two drugs in Da/Dox and SPNs/Da/Dox in the combination group were 1:1. As shown in Figure 4B, the IC50 values of Da, Dox and Da/Dox on BCPAP cells was 2.08, 1.72 and 1.35 μM (for Dox), respectively. These findings indicate the improved synergistic therapeutic efficacy of Da and Dox. In contrast, the Da/Dox combination failed to significantly enhance the killing ability of Dox in L02 cells (Figure 4C). This synergistic effect of Da and Dox on BCPAP cells may have resulted from the inhibitory effect of Da on the proliferation of BRAF V600E positive cells and the pro-apoptotic effect of Dox. Notably, for BCPAP cells, after using supramolecular self-assembled peptides as a carrier, the cell killing abilities of SPNs/Da, SPNs/Dox and SPNs/Da/Dox were significantly stronger than those of the corresponding free drug. Specifically, after encapsulation into SPNs, the IC50 values of Da and Dox decreased 2.1 and 2.13 times, respectively. Accordingly, SPNs/Da/Dox showed the strongest cytotoxic effect on BCPAP cells (Figure 4B). These results may be attributed to cell endocytosis caused by SPNs and the synergy of Da and Dox in BRAF V600E positive cells.

### Mechanism of the synergistic antitumor effect

**Figure 5:**
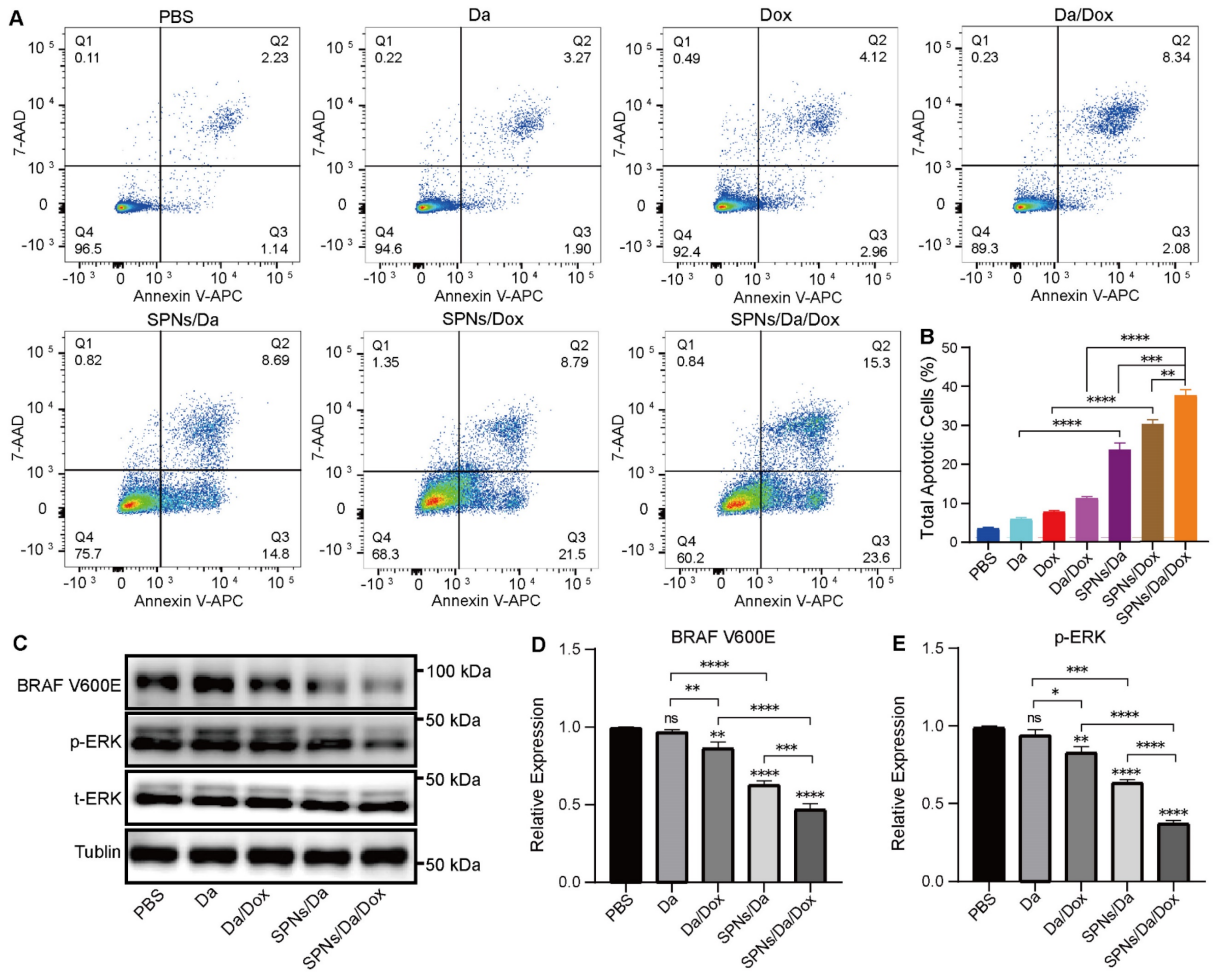
Representative figures (A) and quantitative analysis (B) of apoptosis of BCPAP cells after treated with Da, Da/Dox, SPNs/Da, SPNs/Da/Dox (0.5 µM Da) for 36 h, detected by flow cytometry (*P < 0.05, **P < 0.01, ***P < 0.001, ****P < 0.0001, n = 3). (C) Expression of BRAF V600E and p-ERK in BCPAP cells after treated with Da, Da/Dox, SPNs/Da, SPNs/Da/Dox (0.5 µM Da) for 6 h, determined by Western blot analysis. Quantitative analysis of BRAF V600E (D) and p-ERK (E) determined by Image J (*P < 0.05, **P < 0.01, ***P < 0.001, ****P < 0.0001, n = 3).

Considering that SPNs encapsulation could enhance the killing efficacy of Da and Dox, the mechanism of the synergistic antitumor effect was further studied. According to previous studies, Dox can interfere with the activity of topoisomerase II by inserting it into double-stranded DNA molecules, thereby promoting apoptosis 35. Apoptosis was analyzed by flow cytometry using Annexin V-APC/7-AAD staining (Figure 5A). And the percentages of cells in early, late and total apoptosis were analyzed and listed in Figures 5B and S13. The total apoptosis rates of cells treated with SPNs/Da, SPNs/Dox, and SPNs/Da/Dox for 36 h were 23.49%, 30.29%, and 38.9%, respectively, which were considerably higher than those of the corresponding free drugs (5.17%, 7.08%, and 10.42%). The apoptosis rate of the SPNs/Da/Dox group was the highest owing to the synergistic effects of Da and Dox on cell proliferation and apoptosis and outstanding active targeting of SPNs. Furthermore, the early, late, and total apoptosis exhibited the similar trend in apoptosis. All these results corroborate that SPNs/Da/Dox is a feasible therapeutic strategy for DTC and lay the foundation for the *in vivo* antitumor experiments.

Previous studies have demonstrated that Da can specifically inhibit BRAF V600E via the MAPK signaling pathway and MAPK-ERK cascades in BRAF V600E mutant cells 36-38. To further study the mechanism of Da in SPNs/Da/Dox, the expression of BRAF V600E and p-ERK in BCPAP cells treated with different Da preparations was studied by western blotting. As shown in Figure S14, the expression of p-ERK in BCPAP cells after treatment with SPNs/Da/Dox was reduced to the lowest level at 6 h, which was far lower than that after Da treatment. Accordingly, the expression of BRAF V600E and p-ERK in BCPAP cells treated with Da, Da/Dox, SPNs/Da, SPNs/Da/Dox (0.5 µM Da) for 6 h was further studied. As shown in Figure 5C‒E, Da treatment did not significantly inhibit effect on BRAF V600E or p-ERK. Conversely, Da/Dox and SPNs/Da/Dox decreased the expression levels of these two proteins. In particular, SPNs/Da/Dox reduced the expression of both proteins to the greatest extent. These results suggest that the inhibition of BRAF V600E/p-ERK by SPNs/Da/Dox is more efficient than free drugs in BRAF V600E mutant thyroid cancer cells.

### Biostability of SPNs

In order to attest the biostability, the SPNs was incubated with plasma for different time. As shown in Figures S15 and S16, the peak areas of SPNs (t_R_ = 4.66 min) reduced slightly with the incubation with plasma. Additionally, the corresponding quantitative analysis of stability shows that approximately 88% and 75% of SPNs remained intact after 12 h and 24 h, respectively. Remarkably, approximately 95% of SPNs was virtually intact after incubation with plasma for 6 h. These results demonstrate that SPNs has favorable biostability.

### Targeting ability of SPNs *in vivo*

To verify the targeting capacity induced by RGD ligand, radioactive isotope labeling assay was conducted. SPNs were labeled with ^125^I on the tyrosine residue of the peptide using chloramine-T method. Subsequently, the unreacted ^125^I and other chemicals were removed by radioactive HPLC. The radiochemical purity of the peptides was detected by radioactive TLC scanner, and the result was shown in Figure S17, suggesting the all of free ^125^I was removed. Since the thyroid cell and thyroid cancer cell are capable of absorbing iodine from blood, excessive NaI was intravenously administrated ahead of the injection of Na^125^I to block the sodium/iodide symporter. The blocking effect was shown in Figure S18. Compared with the mouse without the injection of NaI, the radioactive signals in the thyroid tissue and tumor significantly vanished due to the administration of NaI, indicating that the thyroid cell and thyroid cancer cell were blocked. Subsequently, the biodistribution of SPNs was showed in Figure S19. The radioactive signal could be observed in the tumor region (white dash circle) upon the injection of SPNs and reached the maximum at 8 h, after which it declined at 24 h. This indicates that the SPNs could target the tumor by means of RGD ligand.

### *In vivo* synergistic antitumor therapy

**Figure 6:**
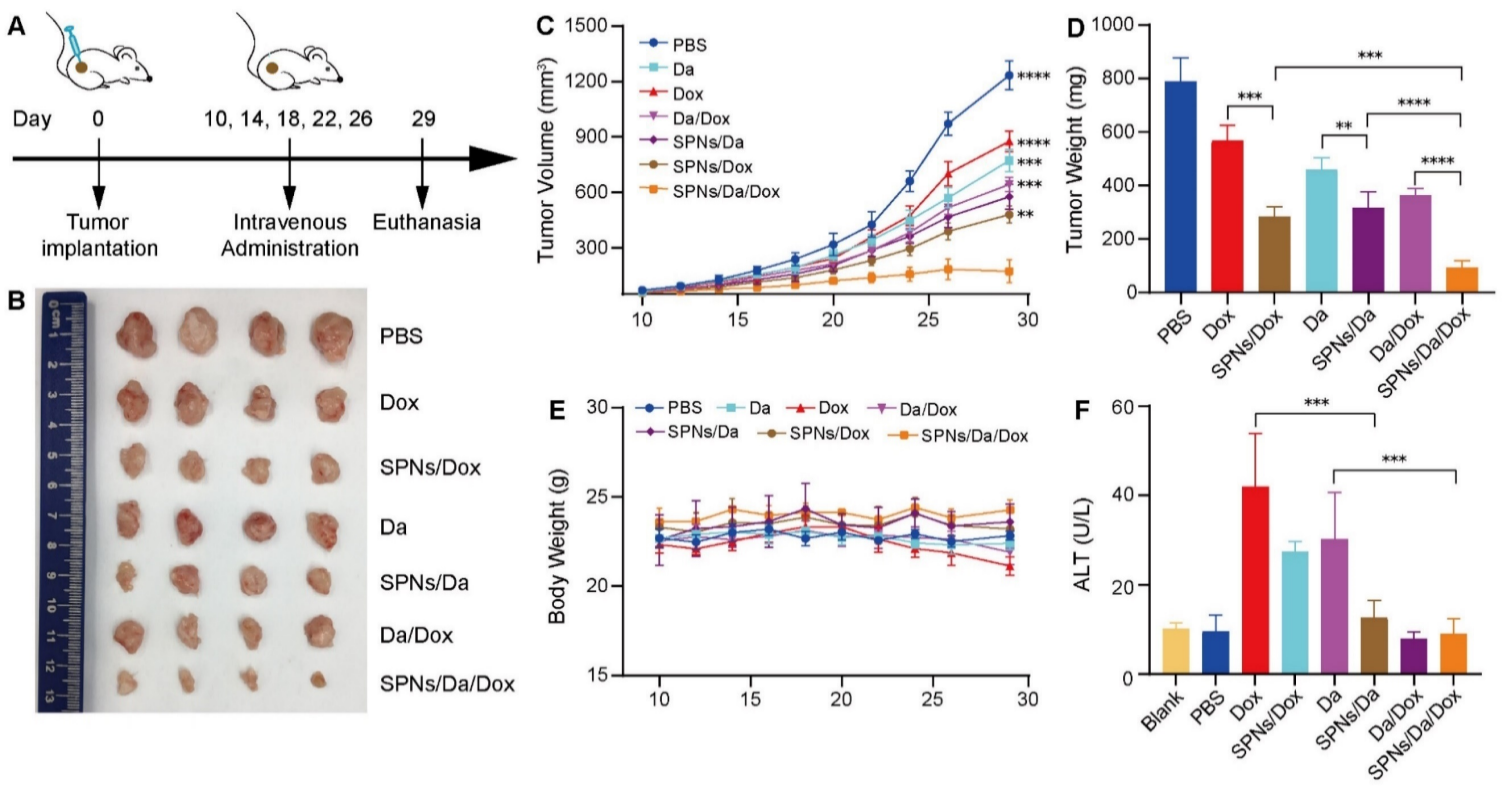
Antitumor activity and biocompatibility of different treatments. (A) Timeline of animal experimental operations. (B) Photographs of tumors collected from mice after varied treatments. (C) Tumor volume curves of tumor-bearing mice during varied treatments as indicated. (D) Average weight of tumors from different groups at the end of the experiment. (E) Average body weight curves of tumor-bearing mice exposed to different treatments (**P < 0.01, ***P < 0.001, ****P < 0.0001, n = 4). (F) Serum glutamic pyruvic transaminase (ALT) level of tumor-bearing mice at the end of the antitumor experiment (***P < 0.001, n = 4).

Motivated by the drug delivery performance and synergistic anticancer efficacy of SPNs/Da/Dox *in vitro*, its antitumor activity *in vivo* in mouse tumor model was subsequently assessed. BCPAP cells were subcutaneously injected into 5-week-old NSG female mice. Tumor-bearing mice with tumor volumes of 50‒75 mm^3^ were randomly divided into seven groups (n = 4). The tumor-bearing mice were then administered Da, Dox, Da/Dox, SPNs/Da, SPNs/Dox, or SPNs/Da/Dox via tail intravenous injection every four days, five times in total (Figure 6A). PBS was used as a control. The course of treatment was 20 days, during which the tumor size and body weight of the mice in each group were measured and recorded every two days. At the end of treatment, all groups of mice were euthanized. Tumors from the mice were dissected and weighed, and pathological sections were prepared for further pathological staining and immunostaining. The main organs, including the heart, liver, spleen, lung, and kidney, were excised and collected for hematoxylin and eosin (H&E) staining. In addition, blood from the mouse eye socket was obtained for biochemical and routine blood analyses.

As shown in Figure 6B‒D, tumor growth was slightly inhibited with Da or Dox therapy compared with the PBS group. Da/Dox showed more efficient inhibition than Da or Dox treatment. The antitumor abilities of SPNs/Da and SPNs/Dox were significantly improved (Figure 6D). Specifically, the tumor weight in SPNs/Da group (317.7 ± 58.3 mg) was significantly smaller than that in Da group (459.6 ± 43.2 mg). The tumor weight in SPNs/Dox group (568.2 ± 56.3 mg) was significantly smaller than that in Dox group (284.7 ± 36.4 mg). Notably, the tumor weight of two mice in the SPNs/Da/Dox group was reduced to less than 90 mg (80.2 and 63.2 mg, respectively), indicative of the best efficacy. Moreover, no obvious variation in body weight was observed in the experimental mice, except for a slight decrease in it after free the Dox treatment (Figure 6E). The weight loss observed in the free Dox treatment could result from the toxicity caused by Dox, which was confirmed by elevated ALT levels (Figure 6F). SPNs/Dox/Da showed excellent biosafety compared to free drugs (Figure S20). These results suggest that SPNs are a safe and promising supramolecular nanoplatform for the tumor-targeted delivery of Da and Dox, which is suitable for synergistic therapy of differentiated thyroid carcinoma.

**Figure 7:**
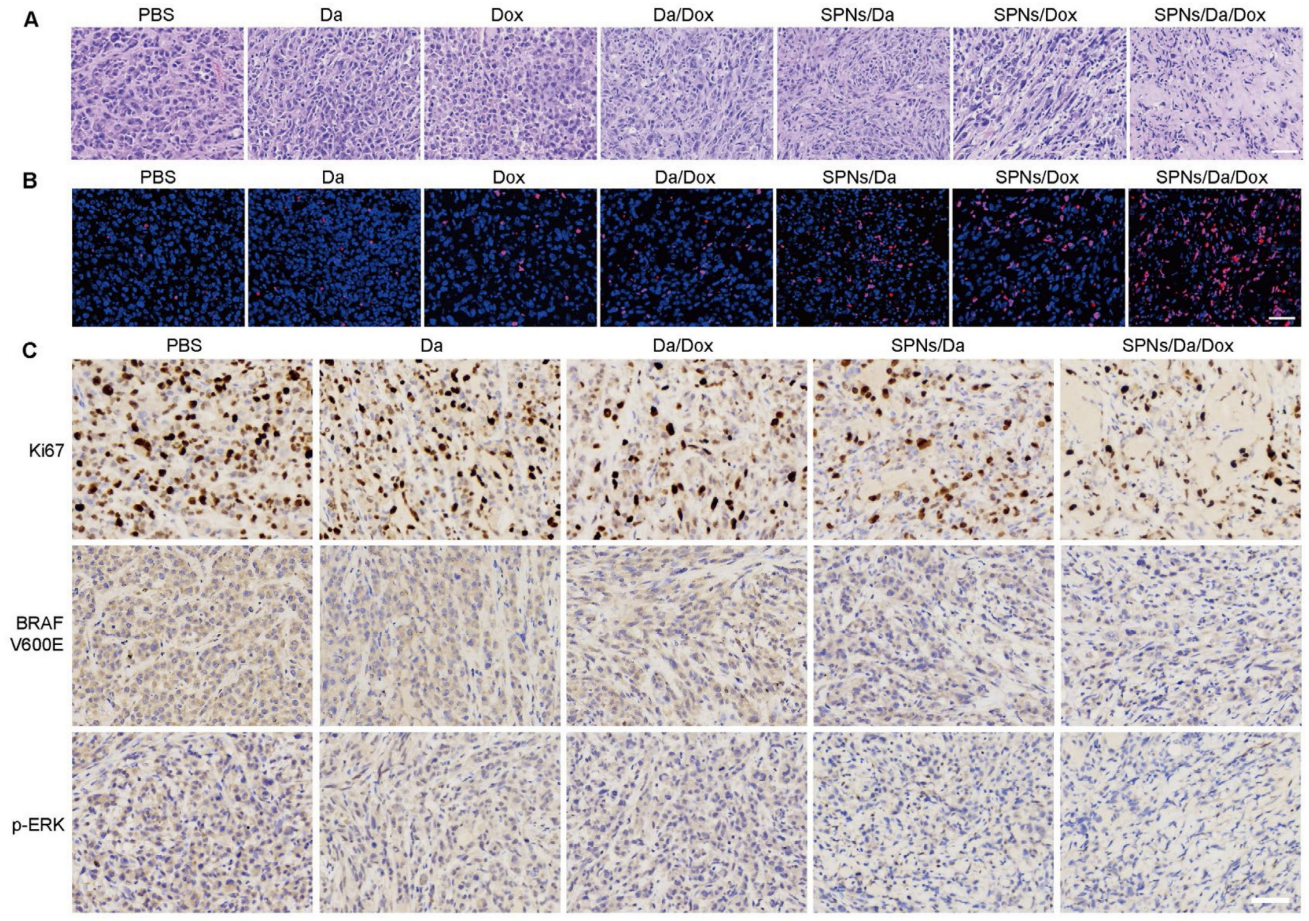
Representative images of hematoxylin and eosin (H&E) (A) and terminal deoxynucleotidyl transferase-mediated deoxyuridine triphosphate nick end labeling (TUNEL) staining (B) of BCPAP tumor exposed to different treatments (scale bar = 50 µm). (C) Immunohistochemical staining of Ki67, BRAF V600E and p-ERK in BCPAP xenografts exposed to different treatments (scale bar = 50 µm).

Subsequently, hematoxylin and eosin (H&E) staining and terminal deoxynucleotidyl transferase-mediated dUTP-biotin nick end labeling (TUNEL) assay were used to assess the antitumor ability at the tumor tissue level. H&E staining images in Figure 7A show that the nuclei were small and sparse after treatment, especially for SPNs/Da/Dox, indicating that tumor viability decreased most significantly after SPNs/Da/Dox treatment. Furthermore, TUNEL immunostaining (Figure 7B) showed that Dox treatment could cause DNA damage in tumors, and its efficacy was significantly enhanced after using SPNs as carriers. Although free Da treatment did not cause obvious DNA damage to the tumor, a certain degree of DNA damage was achieved when encapsulated by SPNs. This is due to the fact that the tumor targeting ability of SPNs significantly enhances the endocytosis of Da and Dox in tumor cells (Figure 3D). The strongest DNA damage occurred in the SPNs/Da/Dox group, resulting in a synergistic effect between the two drugs. These results *in vivo* were consistent with those at the cellular level (Figure 4B).

Since SPNs/Da/Dox can significantly inhibit MAPK-ERK cascades in BCPAP cells, this mechanism in tumor tissue was further investigated by immunohistochemical analysis. Figure 7C shows that, in comparison with high expression of Ki67 in the PBS group and the moderate Ki67 expression in Da, Da/Dox, or SPNs/Da group, SPNs/Da/Dox treatment decreased the expression of Ki67 to the greatest extent. BRAF V600E and p-ERK showed similar expression trends. The results shown in Figure S21 indicate that Dox induced apoptosis in BCPAP xenografts by downregulating the level of anti-apoptotic Bcl-2 and upregulating the levels of pro-apoptotic Bax and Cleaved-Caspase3. Collectively, in terms of the molecular mechanism at the tissue level, the above results once again confirmed the strong targeted inhibitory effect of SPNs-mediated synergistic administration of Da and Dox on BRAF V600E mutant thyroid cancer cells.

## Conclusion

Da constitutes the only BRAF inhibitor approved by the FDA for the treatment of ATC with BRAF V600E^+^, but its therapeutic effect on BRAF V600E^+^ DTC has rarely been studied. Although Dox has been approved for the clinical treatment of radioiodine therapy-refractory and metastatic DTC, its high toxicity limits its clinical application. Nanoformulations of Dox, such as Doxil and Myocet, can efficiently reduce its side effects 39. However, passive targeting of tumor tissue by the enhanced permeability and retention (EPR) effect has a relatively low delivery efficiency 40. Various nanoplatforms have been developed to enhance the synergistic anticancer efficacy of multi-drug combination regimens 41, 42. In this study, we developed a tumor-active targeting supramolecular peptide nanoplatform co-loaded with Da and Dox (SPNs/Da/Dox) for synergistic DTC therapy. *In vitro* cellular uptake experiments demonstrated a much higher cellular uptake efficiency of SPNs/Da/Dox than that of free agents. *In vitro* cytotoxicity and apoptosis detection showed that the killing abilities of Da and Dox were significantly enhanced by encapsulation into SPNs. SPNs/Da/Dox showed targeted killing of cells with high BRAF V600E expression. The strongest synergistic effect of Da and Dox was achieved both *in vivo* and *in vitro* through co-delivery of SPNs. Studies on the molecular mechanism at the cell and tissue levels have indicated that Da and Dox synergistically enhance the killing effect by inhibiting cell proliferation and inducing cell apoptosis. This study verifies the effectiveness of Da in the treatment of BRAF V600E mutant DTC and the highly synergistic effect of Da and Dox co-delivery by tumor-targeted self-assembled peptides. Given that Da has been approved by the FDA for ATC treatment, and Dox is the effective cytotoxic drug for high-risk metastatic DTC, this study opens up a promising paradigm for refractory DTC therapy.

## Supplementary Material

Supplementary figures.

## Abbreviations

DTC: differentiated thyroid carcinoma
PTC: papillary thyroid carcinoma
TSH: thyroid stimulating hormone
RAI: radioiodine therapy
BRAF: V-Raf murine sarcoma viral oncogene homolog B
SPNs: self-assembly peptide nanofibers
Dox: doxorubicin
Da: dabrafenib
p-ERK: ERK phosphorylation
CAC: critical assembly concentration
