# Supplementary material for: Supramolecular nanofibers co-loaded with dabrafenib and doxorubicin for targeted and synergistic therapy of differentiated thyroid carcinoma
Source: Theranostics. 2023 Apr 1;13(7):2140–53. doi: 10.7150/thno.82140 (PMC10157742; doi:10.7150/thno.82140)
Supplement: Supplementary file 1 — Supplementary figures. [file thnov13p2140s1.pdf]

Supporting information

Supramolecular nanofibers co-loaded with dabrafenib and doxorubicin for targeting and synergistic therapy of differentiated thyroid carcinoma

Peng Chen [a], Xiaoyao Cai [b], Ganen Mu [b], Yuansheng Duan [a], Chao Jing [a], Zhimou Yang [c], Cuihong Yang [b,*], Xudong Wang [a,*]

[a] Department of Maxillofacial and Otorhinolaryngological Oncology, Tianjin Medical University Cancer Institute and Hospital, National Clinical Research Center for Cancer, Key Laboratory of Cancer Prevention and Therapy, Tianjin's Clinical Research Center for Cancer, Tianjin 300060, China

[b] Key Laboratory of Radiopharmacokinetics for Innovative Drugs, Chinese Academy of Medical Sciences, Tianjin Key Laboratory of Radiation Medicine and Molecular Nuclear Medicine, Institute of Radiation Medicine, Chinese Academy of Medical Sciences & Peking Union Medical College, Tianjin 300192, China

[c] State Key Laboratory of Medicinal Chemical Biology, College of Life Sciences, Key Laboratory of Bioactive Materials, Ministry of Education, Collaborative Innovation Center of Chemical Science and Engineering, and National Institute of Functional Materials, Nankai University, Tianjin 300071, China.

[*] Corresponding authors.

E-mail addresses: Dr. Xudong Wang (wxd.1133@163.com) and Dr. Cuihong Yang (yangcuihong@irm-cams.ac.cn)

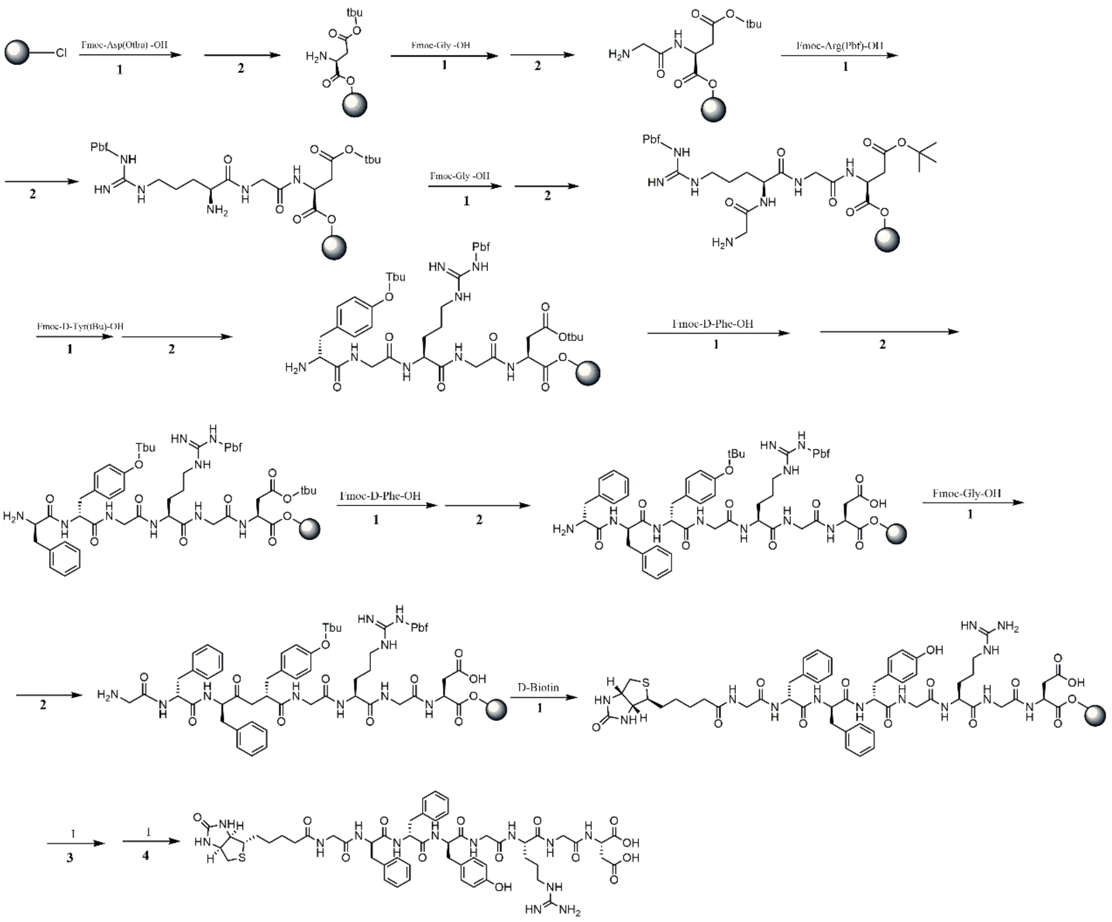

1. HBTU(2 eq)/DIEA(4 eq), RT, 2 h
2. Piperidine/DMF (20%), 30 min
3. TFA/TIS/H_2O=95%:2.5%:2.5%, 30 min
4. Et_2O, overnight

Figure S1. Synthetic routes and molecular structure of Biotin-G^{D}F^{D}F^{D}YGRGD.

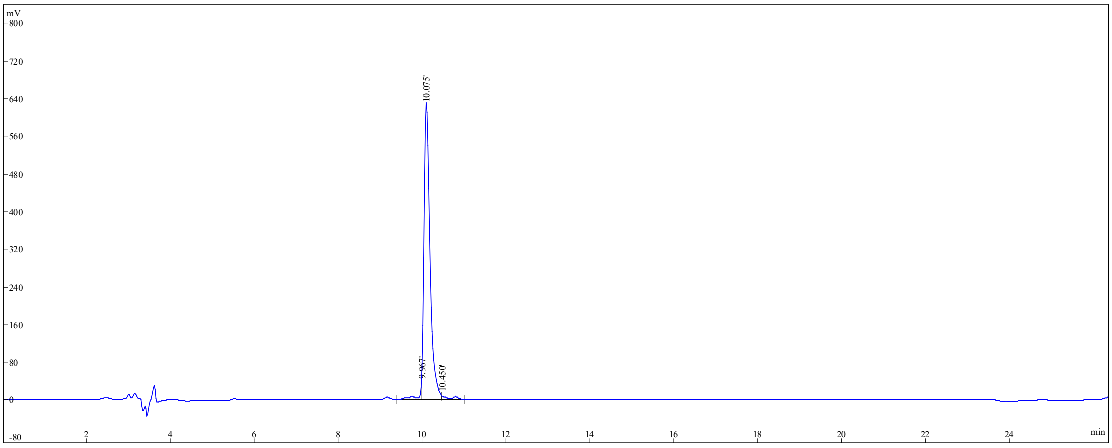

Figure S2. HPLC chromatogram of Biotin-G^{D}F^{D}F^{D}YGRGD

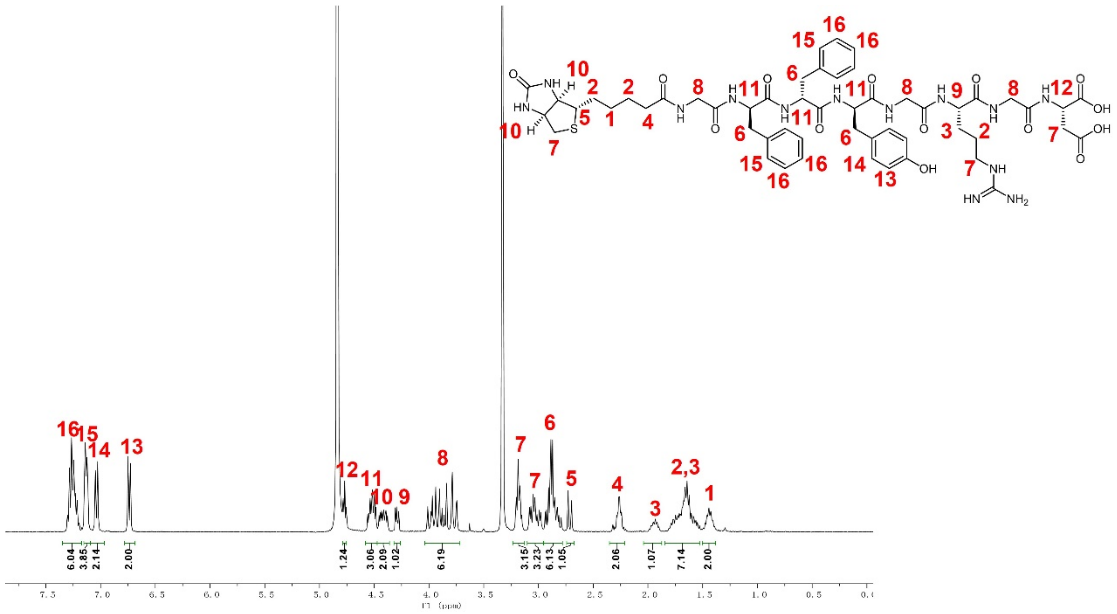

Figure S3. ^{1}H NMR of Biotin-G^{D}F^{D}F^{D}YGRGD. 1H NMR (400 MHz, Methanol-d4) δ 7.35–7.17 (m, 6H), 7.13 (dd, J = 7.2, 2.5 Hz, 4H), 7.04 (d, J = 8.4 Hz, 2H), 6.74 (d, J = 8.4 Hz, 2H), 4.77 (t, J = 5.8 Hz, 1H), 4.52 (ddd, J = 15.6, 8.5, 5.2 Hz, 3H), 4.42 (ddd, J = 16.3, 8.8, 5.4 Hz, 2H), 4.29 (dd, J = 7.9, 4.4 Hz, 1H), 4.04–3.72 (m, 6H), 3.18 (q, J = 6.6 Hz, 3H), 3.10–2.96 (m, 3H), 2.95–2.78 (m, 6H), 2.71 (d, J = 12.8 Hz, 1H), 2.35–2.21 (m, 2H), 1.93 (dd, J = 13.8, 7.3 Hz, 1H), 1.85–1.52 (m, 7H), 1.45 (dd, J = 14.0, 7.1 Hz, 2H).

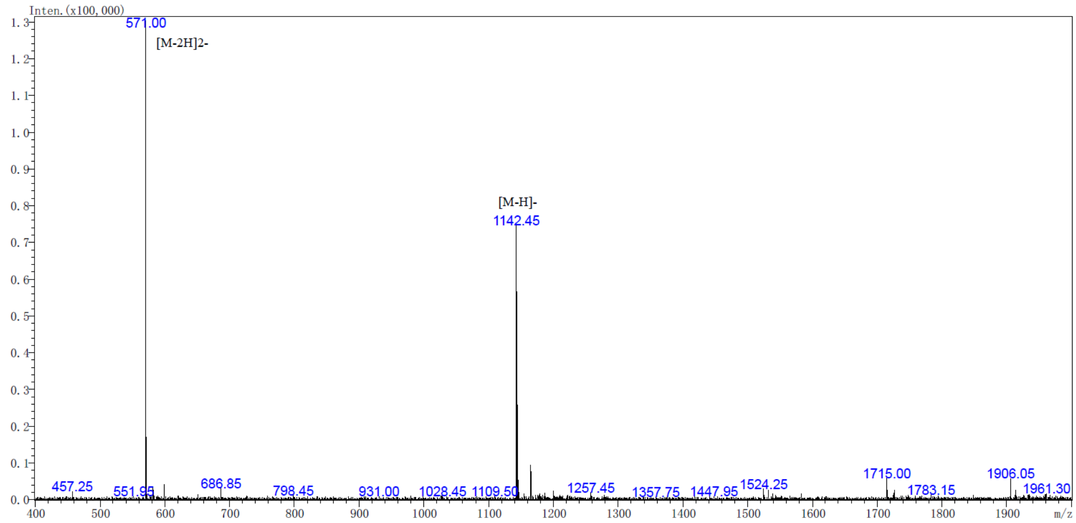

Figure S4. Mass spectrum of Biotin-G^{D}F^{D}F^{D}YGRGD.

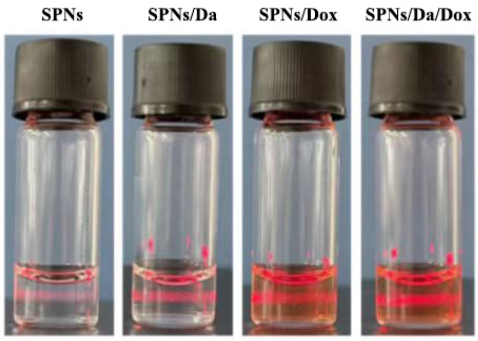

Figure S5. Tyndall effect images of self-assembled SPNs, SPNs/Da, SPNs/Dox, SPNs/Da/Dox.

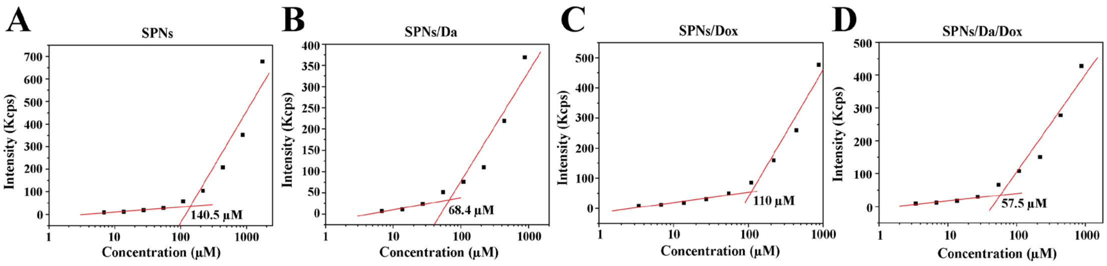

Figure S6. Critical assembly concentration (CAC) of SPNs, SPNs/Da, SPNs/Dox, SPNs/Da/Dox.

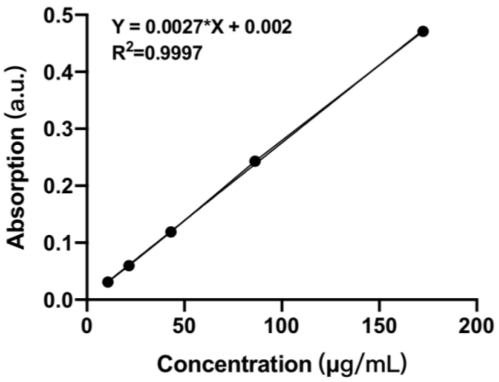

Figure S7. The standard curve of absorption as a function of Dox concentration in solutions.

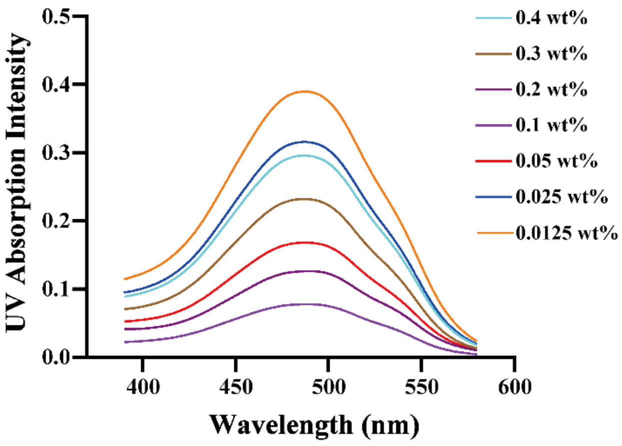

Figure S8. The UV–vis absorbance spectra of the supernatant of SPNs/Dox co-assemblies after ultracentrifugation (0.5 mM Dox and different concentrations of SPNs).

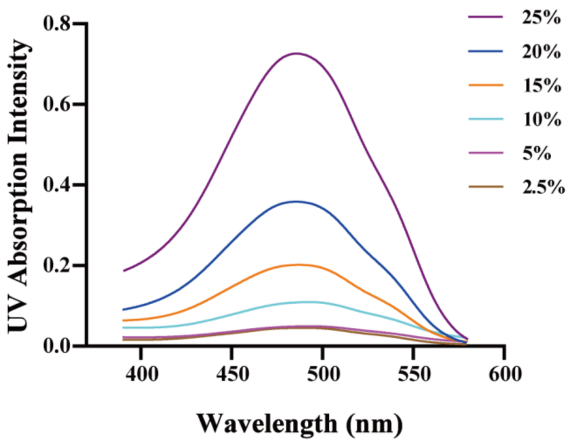

Figure S9. The UV–vis absorbance spectra of the supernatant of SPNs/Dox after ultracentrifugation (0.1 wt% SPN

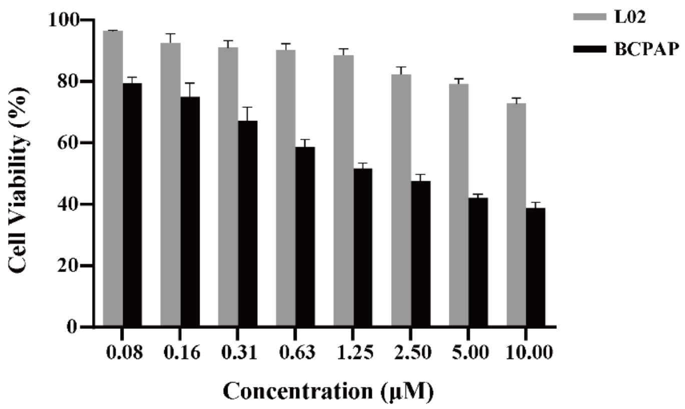

Figure. S10. The viability of BCPAP and L02 cells treated by Da for 48 h (n = 3).

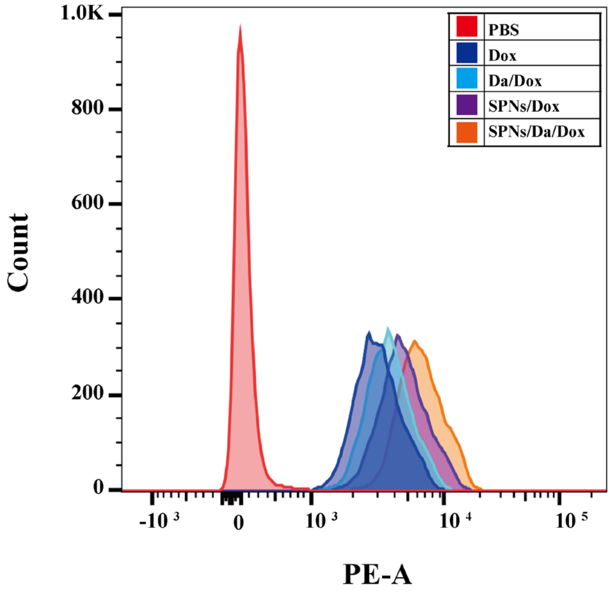

Figure S11. Flow cytometry fluorescence spectra of BCPAP cells treated with different formulations for 4 h.

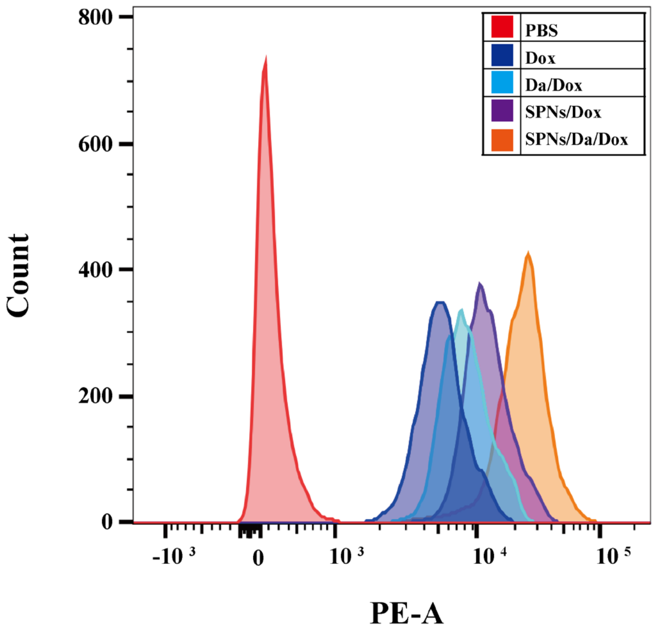

Figure S12. Flow cytometry fluorescence spectra of BCPAP cells treated with different formulations for 12 h.

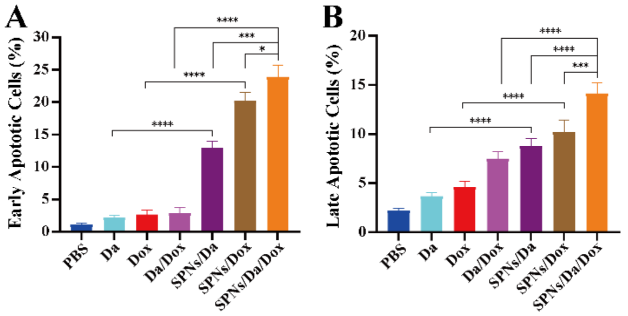

Figure S13. Quantitative analysis of early apoptosis (A) and late apoptosis (B) of BCPAP cells after treated with Da, Da/Dox, SPNs/Da, SPNs/Da/Dox (0.5 μM Da) for 6 h, detected by flow cytometry. (**P*<0.05, ****P*<0.001,*****P*<0.0001, n = 3)

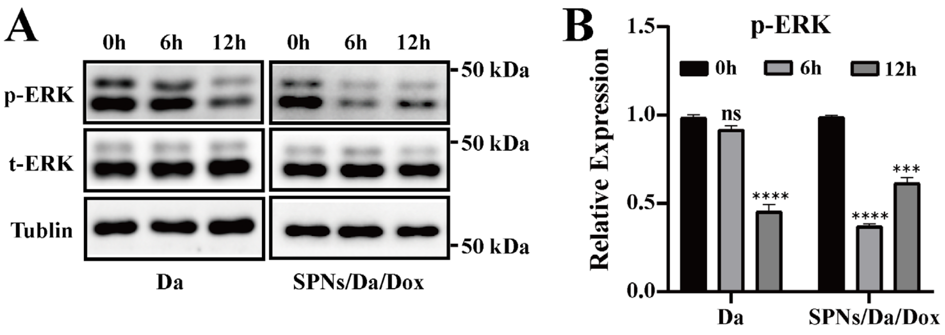

Figure S14. (A) Expression of p-ERK in BCPAP cells exposed to Da or SPNs/Da/Dox for 0 h, 6 h and 12 h determined by Western blot analysis. (B) Quantitative analysis of p-ERK expression determined by Image J (n = 3).

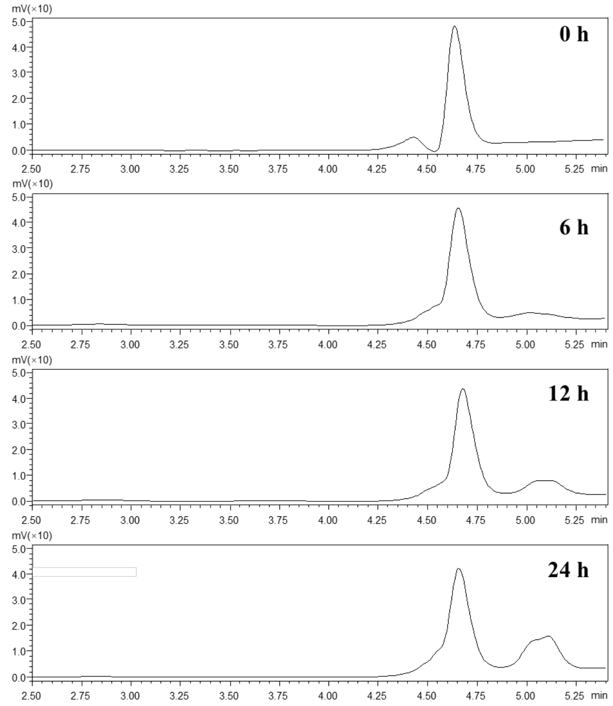

Figure S15. LC-MS traces of SPNs incubated with plasma at different time points.

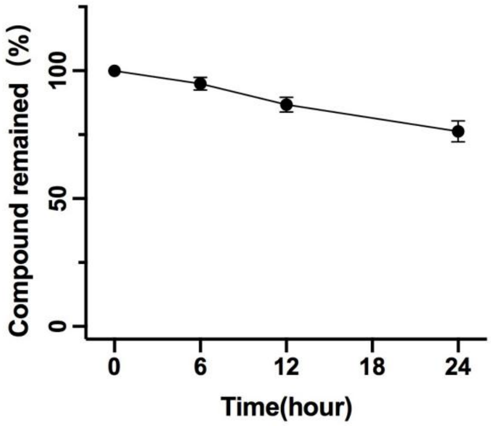

Figure S16. Quantitative analysis of stability of SPNs incubated with plasma at different time points (n = 3).

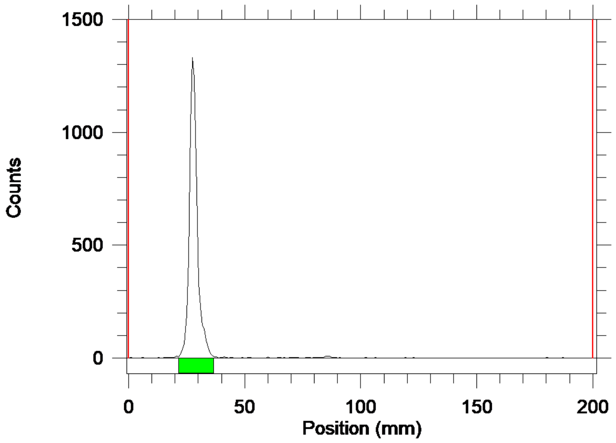

Figure S17. TLC chromatogram of ^{125}I-labeled SPNs in 1 min.

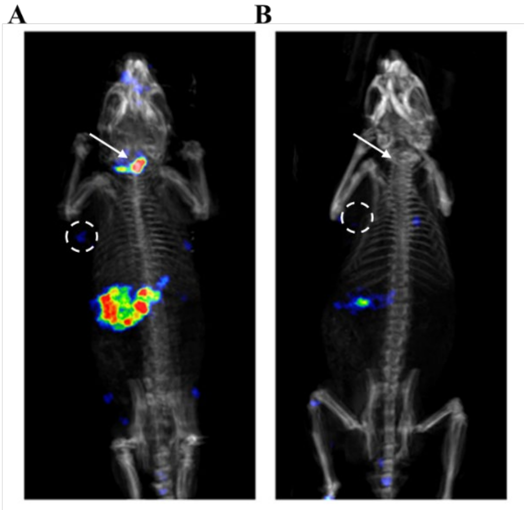

Figure S18. The Gamma-camera images of (A) mouse only injected with Na^{125}I and (B) mouse sequentially injected with NaI and Na^{125}I. White dash circle indicates the location of thyroid cancer xenograft, and the white arrow indicates thyroid.

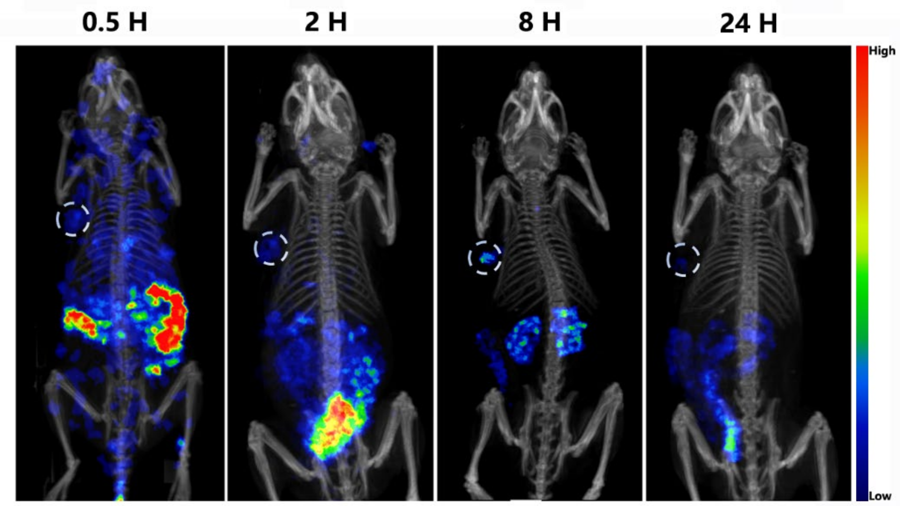

Figure S19. The representative Gamma-camera images of mice injected with ^{125}I-labeled SPNs at 0.5, 2, 8 and 24 h. White dash circle indicates the location of thyroid cancer xenograft.

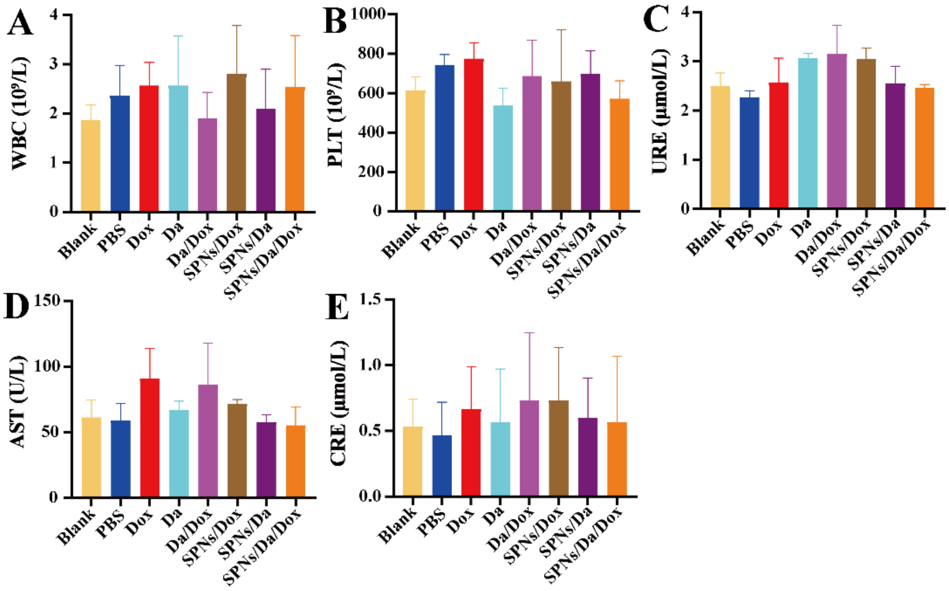

Figure S20. Blood biochemistry data of mice in different groups (n = 4).

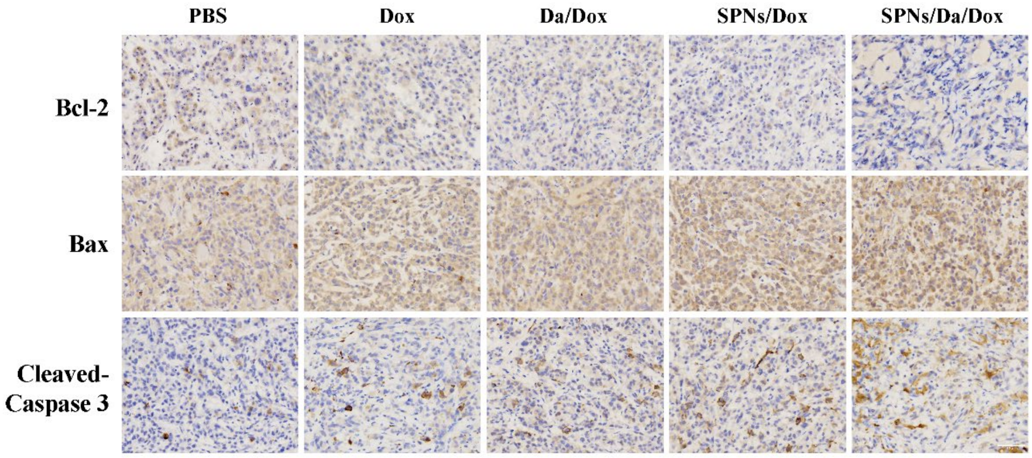

Figure S21. Representative immunohistochemical staining of Bcl-2, Bax and Cleaved-Caspase 3 in BCPAP xenografts exposed to different treatments (scale bar = 50 μm).
